# Is a Hospital Pageant Practicable?

**Published:** 1921-05-21

**Authors:** Ralph B. Cannings

**Affiliations:** City of London Maternity Hospital, City Road, E.C. 1.


### IS A HOSPITAL PAGEANT PRACTICABLE?

*To the Editor of* THE HOSPITAL.

Sir,—The idea of a Pageant is, I think, a good one, but to carry it out successfully requires considerable preparation. I fear it would be hopeless to try to perfect arrangements by June 26, especially as the holiday season is upon us. I note that the Great Northern Central Hospital Secretary has suggested a meeting, so that views on the matter will doubtless be aired.—Yours faithfully,

Ralph B. Cannings.
City of London Maternity Hospital, City Road, E.C. 1.

